# Atherosclerotic Plaque Characterization Magnetic Resonance Imaging In Vitro at 1.5 Tesla for the Assessment of Coronary Artery Disease

**DOI:** 10.3390/jcm15093507

**Published:** 2026-05-03

**Authors:** Angelika Myśliwiec, Dawid Leksa, Avijit Paul, Marvin Xavierselvan, Adrian Truszkiewicz, Dorota Bartusik-Aebisher, David Aebisher

**Affiliations:** 1 Department of Biochemistry and General Chemistry, Medical Faculty, Collegium Medicum, University of Rzeszów, 35-959 Rzeszów, Poland; amysliwiec@ur.edu.pl (A.M.); dbartusikaebisher@ur.edu.pl (D.B.-A.); 2 Rzeszów Center for Vascular and Endovascular Surgery, 35-310 Rzeszów, Poland; dleksa@gmail.com; 3 Department of Biomedical Engineering, Tufts University, Medford, MA 02155, USA; avijit.paul@tufts.edu (A.P.); marvin.xavierselvan@tufts.edu (M.X.); 4 Department of Photomedicine and Physical Chemistry, Medical Faculty, Collegium Medicum, University of Rzeszow, 35-959 Rzeszów, Poland; atruszkiewicz@ur.edu.pl

**Keywords:** atherosclerotic plaque, atherosclerotic, MRI, T1, T2, atherogenesis

## Abstract

**Background/Objectives:** The composition of atherosclerotic plaques is increasingly recognized as a key factor determining cardiovascular risk. Features such as intraplaque hemorrhage, a necrotic lipid core, and the integrity of the fibrous cap are strongly associated with plaque instability and the occurrence of adverse clinical events. Magnetic resonance imaging (MRI) allows for non-invasive characterization of plaque microstructure through quantitative mapping of T1 and T2 relaxation times; however, image noise may limit the accuracy of these measurements. **Methods:** In this experimental study, a total of 15 ex vivo atherosclerotic plaque samples were imaged using a 1.5T scanner with a fast spin-echo sequence featuring variable repetition times (TR: 200–12,000 ms) and echo times (TE: 21–240 ms) to obtain T1 and T2 maps. An Attention–Residual–Dense U-Net neural network was trained on pairs of noisy and reference images to reduce Rician noise while preserving structural details. **Results:** The 15 samples examined exhibited T1 values ranging from 1768 to 3294 ms and T2 values ranging from 138 to 202 ms, which were shorter than those for water (T1: 3323 ms; T2: 114 ms), which is consistent with the presence of collagen, lipids, and mineral deposits. Variability among samples reflected differences in composition, with the shortest relaxation times suggesting advanced calcifications. The application of deep learning methods allowed for a threefold improvement in the signal-to-noise ratio (SNR) while preserving the microarchitecture of the lamina. **Conclusions:** Quantitative T1/T2 mapping combined with deep learning-based image enhancement methods constitutes a robust tool for high-resolution characterization of atherosclerotic plaque composition under ex vivo conditions. The results obtained indicate the potential for translating this method to in vivo studies to better detect tissue heterogeneity and features associated with plaque instability.

## 1. Introduction

Modern atherosclerosis diagnostics employs both invasive and non-invasive methods to assess the severity of the disease and cardiovascular risk. The most commonly used non-invasive techniques include computed tomography (CT), nuclear medicine scans, magnetic resonance imaging (MRI), exercise testing, and analysis of biochemical biomarkers [1,2].

**Figure 1:**
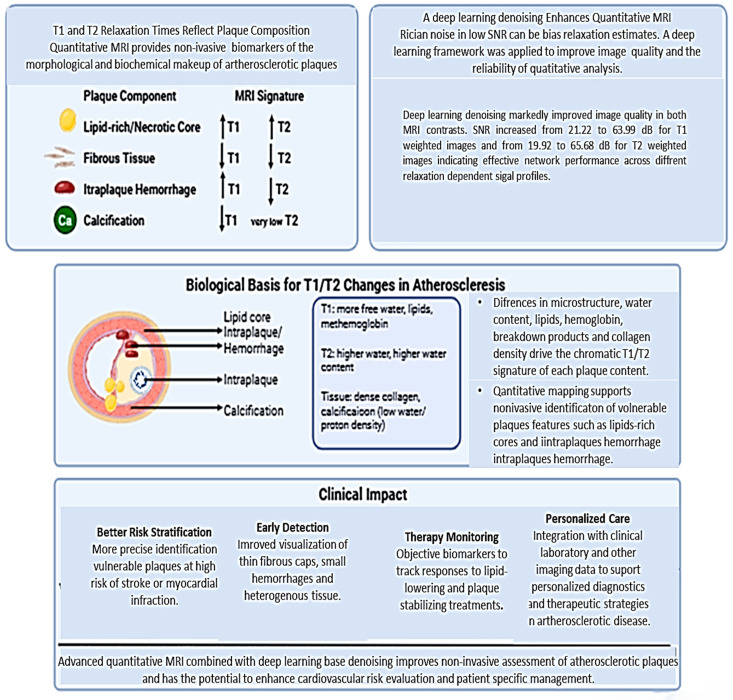
A diagram summarizing the key findings.

MRI allows for the differentiation of tissue components, such as water, lipids, and fibers, through the use of various imaging contrasts. This method is particularly effective in assessing large, immobile vessels (e.g., carotid arteries), whereas imaging of the coronary arteries remains challenging due to their small diameter and mobility [3]. MRI also allows for the assessment of plaque instability features, such as fibrous cap thickness or the presence of intraplaque hemorrhage (Figure 1) [4,5].

In recent years, advances in quantitative mapping of T1 and T2 relaxation times have enabled more precise, non-invasive characterization of the composition of atherosclerotic plaques. These parameters reflect the physicochemical properties of tissues and correlate with histological features, such as the presence of lipids, calcifications, fibrosis, or inflammatory processes, allowing for the differentiation of stable and rupturable plaques [6,7].

High-resolution MRI techniques, utilizing multicontrast sequences (T1, T2, PD, contrast-enhanced imaging), achieve high sensitivity and specificity in identifying key plaque components, such as the lipid necrotic core, intra-plaque hemorrhage, or damage to the fibrous cap [8,9,10,11,12]. The results of these studies show good agreement with histopathological analysis and underscore the importance of MRI as a tool in cardiovascular risk assessment.

**Figure 2:**
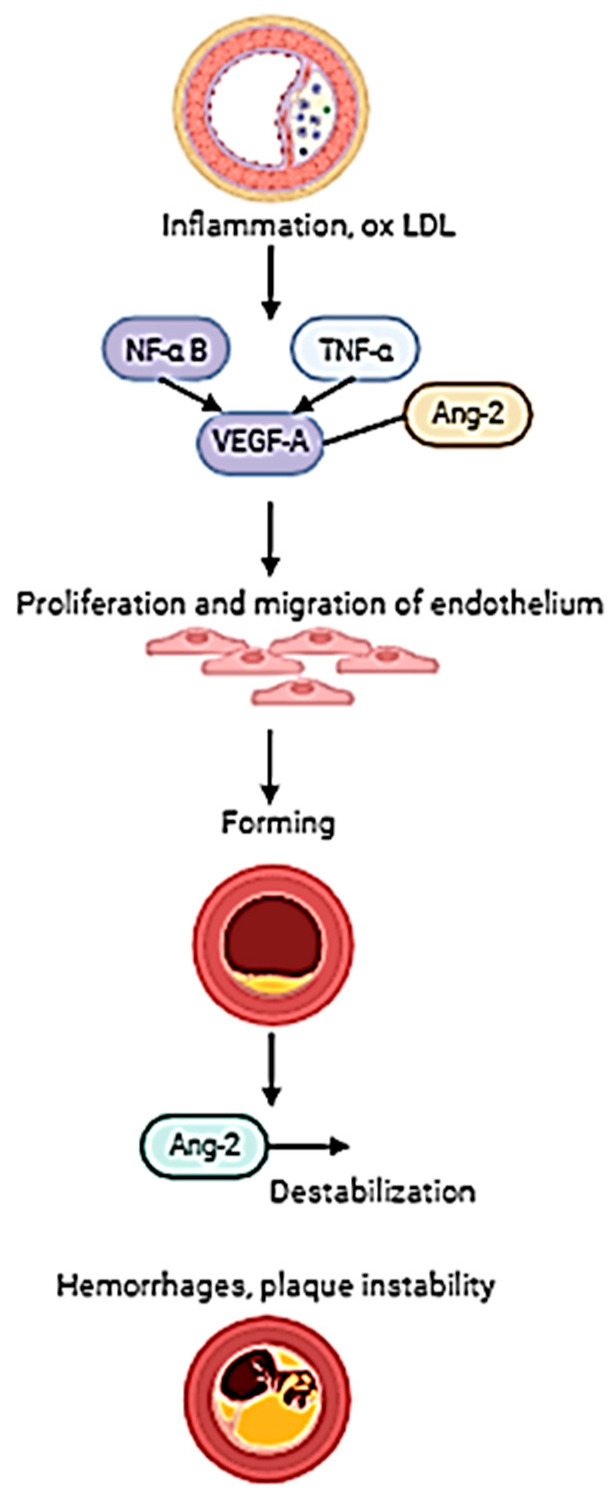
Pathological angiogenesis in atherosclerotic plaques. Inflammation and oxidized LDL (oxLDL) within atherosclerotic lesions activate signaling cascades involving NF-κB, TNF-α, and angiopoietin-2 (Ang-2), leading to VEGF-A upregulation. This promotes endothelial proliferation and migration, resulting in the formation of immature neovessels. Unlike physiological angiogenesis, these vessels lack pericyte stabilization and are prone to Ang-2-mediated destabilization, leading to intraplaque hemorrhage and accelerated plaque instability [19].

Atherosclerosis is a chronic inflammatory–metabolic process leading to the formation of plaques composed of a lipid core and a fibrous cap [13]. Their instability is associated with the presence of inflammation, neovascularization, and degradation of the extracellular matrix, involving, among others, metalloproteinases such as MMP9 (Figure 2) [14,15,16,17,18].

The aim of this study is to apply quantitative T1 and T2 mapping in combination with an advanced deep learning-based noise reduction method (Attention–Residual–Dense U-Net) to improve the accuracy of characterizing the composition of atherosclerotic plaques. This approach enables a more reliable representation of the physicochemical properties of tissues and reduces the impact of noise on the estimation of relaxation parameters.

The innovative nature of this work lies in the integration of quantitative MRI imaging with deep learning methods in the analysis of ex vivo samples, allowing for a more precise and reproducible assessment of plaque microstructure compared to qualitative approaches. Additionally, the strategy used to improve the signal-to-noise ratio enhances the sensitivity of detecting subtle tissue differences, representing a significant step toward translating these methods to in vivo studies and potential clinical applications.

## 2. Materials and Methods

### 2.1. Samples of Atherosclerotic Plaques

This study was approved by the Bioethics Committee of the Medical Chamber in Rzeszów (No. 43/2024/B), which ensures that the procedures for collecting samples and the use of patient data complied with ethical standards for scientific research. A total of 15 ex vivo atherosclerotic plaque samples were collected from 15 different patients undergoing carotid endarterectomy. All patients provided informed consent, collected from male patients, to participate in this study and for the use of tissue samples for scientific purposes.

Samples were collected from 15 patients (n = 15) diagnosed with advanced carotid atherosclerosis requiring surgical treatment. The endarterectomy procedure was performed in the clinical context of significant vascular stenosis associated with the presence of cardiovascular risk factors, such as hypertension and hyperlipidemia.

Tissue samples were collected during endarterectomy, a procedure involving the removal of atherosclerotic plaques from the interior of the artery, which allowed for the acquisition of material of sufficient quality and quantity for further studies. The study protocol, covering both the sampling methodology and MRI imaging procedures, was approved by the relevant institutions.

**Figure 3:**
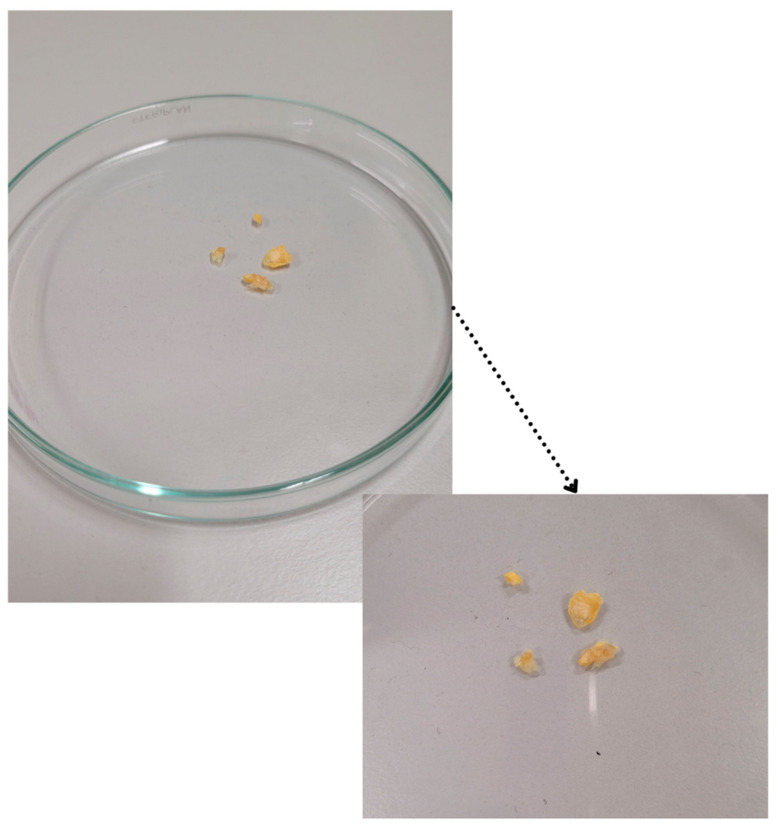
Gross appearance of atherosclerotic plaque specimens obtained by carotid endarterectomy. Fragments are collected from patients with diagnosed atherosclerosis (n = 15). Main panel shows specimens in a glass petri dish; inset provides magnified view of the yellowish-white calcified plaque material. Samples were immediately frozen at −80 °C following collection to preserve tissue integrity for subsequent MRI analysis.

MRI measurements were performed in vitro on tissues taken from the patients (ex vivo). This approach allows for high-resolution images and precise data on T1 and T2 parameters, as it eliminates interference related to tissue movement, blood pulsation, or the influence of surrounding anatomical structures. After collection, samples were immediately frozen at −80 °C to halt metabolic processes and preserve tissue structure. Samples were stored under appropriate conditions until use. Prior to imaging, tissues were thawed at room temperature for approximately 5 h without additional thermal procedures to avoid damaging tissue structure (Figure 3).

Immersion of samples in water was an integral part of the experimental protocol and was intended to ensure repeatable and physiologically relevant measurement conditions during MRI acquisition. During sample preparation, each excised atherosclerotic plaque was placed in a tightly sealed, non-magnetic container filled with distilled water (or PBS buffer, if you want a more “biological” version) so that the tissue was completely submerged and contained no air bubbles in its immediate vicinity.

Immersion in water served several key functions: first, it prevented tissue dehydration during long relaxation sequences, which could significantly alter T_1_ and T_2_ relaxation times; second, it minimized differences in magnetic susceptibility at the tissue–air interface, which reduced artifacts and improved magnetic field homogeneity; third, water provided a reference environment with well-known relaxation times, allowing for control of measurement stability and assessment of relative changes in T_1_ and T_2_ in the samples under study.

The effect of immersion in water on T_1_ and T_2_ relaxation times was taken into account in the analysis by parallel measurements of the signal in the area of water surrounding the sample, which allowed for a comparison of tissue relaxation values relative to the reference medium. This made it possible to distinguish between changes resulting from the actual composition of the atherosclerotic plaque and potential environmental effects related to the measurement conditions.

**Figure 4:**
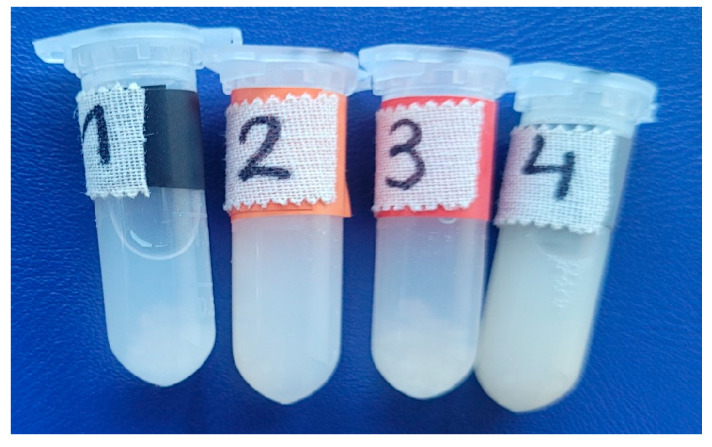
Prepared atherosclerotic plaque samples in Eppendorf tubes prior to MRI acquisition. An example of selected samples from a total of 15 samples obtained from different patients are presented; they were immersed in distilled water and positioned for imaging. Sample masses ranged from 0.0321 g to 0.2462. Samples were thawed at room temperature for approximately 5 h before measurement to ensure uniform temperature (21 °C) during MRI acquisition.

Samples were prepared and subjected to further analysis, including MRI to assess changes in T1 and T2 relaxation times, which allows detailed examination of tissue structure in the context of atherosclerosis. MRI measurements of the samples were taken 3 times for each sample (Figure 4).

### 2.2. MRI Analysis

Spin-net (T1) and spin-spin (T2) relaxation times were measured using an advanced Tesla Optima MR360 MRI system, manufactured by General Electric Healthcare (Milwaukee, WI, USA), with dedicated SV23 GE MR Service Pack software. The system is equipped with high-quality imaging technology, enabling the detailed and precise results needed to analyze tissue relaxation times. Imaging was performed using a fast spin echo (FSE) sequence in an axial projection, which allows for clear and resolved images in the cross sections required for further analysis. The use of a small elastic coil contributes to the precision of the measurements, allowing accurate imaging within small structures such as atherosclerotic plaques.

**Figure 5:**
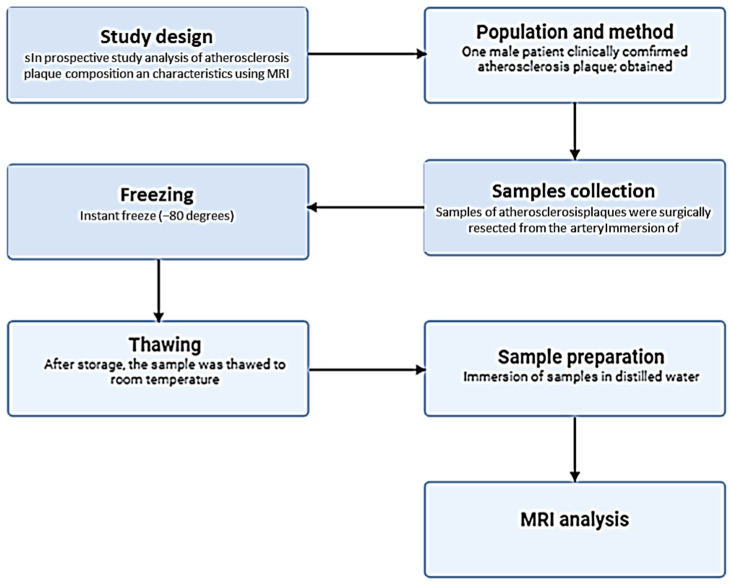
Schematic overview of the experimental workflow. Atherosclerotic plaque specimens were obtained by surgical resection (endarterectomy) from ex vivo samples of atherosclerotic plaques from 15 patients with clinically confirmed atherosclerosis. Samples were immediately frozen at −80 °C for preservation and then thawed to room temperature prior to preparation. Samples were immersed in distilled water and subjected to quantitative T_1_ and T_2_ relaxation mapping using a 1.5T MRI system.

During the study, DICOM images were analyzed, which allowed the evaluation of tissue structure and the acquisition of ROI (region of interest) measurements, i.e., isolated areas for analysis within the images obtained from the series of scans performed for each test. With this data, it was possible to determine T1 and T2 relaxation times, which are key parameters that characterize the physicochemical properties of tissues, such as the content of water, lipids, proteins and other internal structures (Figure 5).

Throughout the experiment, the technical parameters of MRI, such as a scanning matrix of 320 × 224, a section thickness of 4 mm, and a NEX (number of repetitions) of 1.0, remained constant, ensuring the uniformity of the examination conditions and allowing comparison of results between different stages of the experiment. These parameters are important because they directly affect the quality of the images and the accuracy of the results obtained. T1 measurements were carried out in 12 steps with varying repetition time (TR), which ranged from 200 ms to 12,000 ms. The echo time (TE) was set to a fixed value of 27 ms. For T2 measurements, 12 steps were also taken but with the TR repetition time set to 10,000 ms and the echo time (TE) varying from 21 ms to 240 ms. The TR and TE values are key to obtaining appropriate results depending on the specific tissue under study, which in this case allows accurate assessment of relaxation times within atherosclerotic plaques.

The MRI examinations were performed using a 1.5 T magnetic field strength device, which is in line with commonly used clinical standards. A 1.5 T magnetic field provides good image quality, wide availability of equipment, and reliable diagnostic performance, also enabling the assessment of small tissue structures such as atherosclerotic plaques.

Imaging was performed in vitro on tissues collected from patients, maintaining a stable sample temperature of 21 °C. Controlled temperature is important because T1 and T2 relaxation times depend on temperature, and its stability ensures repeatability and reliability of measurements.

The whole process of MRI with uniform examination conditions is an important element in analyzing the progress of atherosclerosis treatment and in evaluating the effectiveness of therapy, which may contribute to the further development of diagnostic methods used in clinical practice.

### 2.3. Deep Learning Architecture

#### 2.3.1. Noise Modeling and Dataset Preparation

MRI magnitude images are affected by Rician-distributed noise arising from Gaussian noise in the real and imaginary channels of the complex MR signal [20,21,22]. To realistically simulate clinical acquisition conditions, clean reference MRI images were corrupted with synthetic Rician noise at varying noise levels. These paired datasets (noisy input, clean target) enabled supervised learning while maintaining full control over the ground truth signal. All images were normalized to the range [0, 1] and stored as single-channel (grayscale) PNG files. The dataset was randomly split into training (80%), validation (10%), and testing (10%) subsets at the subject level to avoid data leakage. The noise was modeled with the following equation:

$$p_{M}\left(M\right)\approx \frac{1}{\sqrt{2\pi \sigma^{2}}}e^{\frac{{-(M-\sqrt{A^{2}+\sigma^{2}})}^{2}}{{2\sigma }^{2}}}$$

A is the image pixel intensity for a clean image, whereas M is the intensity for a Rician noise-corrupted image. MRI training and validation dataset was taken from the publicly available dataset: https://www.kaggle.com/datasets/masoudnickparvar/brain-tumor-mri-dataset (accessed on 15 December 2025).

#### 2.3.2. Network Architecture

Denoising was performed using a custom-designed Advanced Attention–Residual–Dense U-Net, combining multiple architectural principles: Residual blocks to stabilize training and preserve low-frequency anatomical information [23]; dense connectivity to promote feature reuse and enhance gradient flow; attention gates in the decoder to suppress irrelevant background regions and focus on anatomically meaningful plaque structures; and multi-scale encoder–decoder design to capture both global context and fine structural details. The network operates on 256 × 256 MRI slices with a single input channel and outputs a denoised image of identical resolution.

#### 2.3.3. Loss Function and Optimization

Training employed a composite loss function combining:

L1 loss, enforcing pixel-wise fidelity and reducing bias in homogeneous regions.

Structural Similarity Index Measure (SSIM) loss, promoting perceptual and structural preservation critical for medical interpretation [22].

The total loss was defined as:

$$L={\left\|y_{true}-y_{pred}\right\|}_{1}+0.5\left(1-SSIM(y_{true},y_{pred})\right)$$

Optimization was performed using the Adam optimizer (learning rate = 1 × 10^−4^). Early stopping, learning-rate scheduling, and model checkpointing were used to prevent overfitting and ensure convergence. The model was trained for up to 100 epochs with mini-batches of example images. During inference, denoised MRI images were generated for the independent test set and subsequently used for quantitative analysis and visualization. All experiments were implemented in TensorFlow v2.16.1/Keras.

#### 2.3.4. Image Quality Metrics

SNR: SNR was determined as a ratio of actual signal from the object to the background noise as per the formula in [22]:

$$SNR=10\cdot log\frac{mean(ROI)}{\sigma (bg)}$$

ROI is region of interest containing signals, bg is background, and σ(*) is the standard deviation.

### 2.4. Statistical Analysis

A statistical analysis was conducted to assess the effect of water immersion on T1 and T2 relaxation times in samples taken from atherosclerotic plaques. The unit of analysis was the relaxation values (T1 and T2) determined within defined regions of interest (ROI). This study included 15 samples (n = 15), each from a different patient, which eliminates inter-subject dependencies.

At the same time, it should be noted that multiple ROIs were analyzed within each sample, introducing a potential nested data structure (ROI measurements nested within the sample). However, this study adopted an exploratory and descriptive approach, focused primarily on the quantitative characterization of changes in relaxation parameters and their comparison across samples. Consequently, formal statistical models accounting for hierarchical data structures, such as mixed-effects models or multilevel models, were not used.

This decision was primarily driven by the limited sample size at the higher level (n = 15) and the biological heterogeneity of the material, which could lead to unstable parameter estimates in hierarchical models. Instead, the analysis was conducted at the ROI measurement level, treating the obtained values as observations describing local tissue properties, while exercising interpretive caution. In particular, it is emphasized that the potential correlation between measurements within the same sample was not explicitly modeled, which may affect the effective degrees of freedom and the variance of the estimators. Consequently, the results obtained should be interpreted as exploratory characterizations of trends and relationships, rather than as a basis for formal population-level inference.

All T1 and T2 measurements were processed and analyzed using MATLAB 23.2 software (MathWorks, Natick, MA, USA). Relaxation curves were fitted using the nonlinear least squares method, which enabled the precise determination of relaxation times within the designated ROIs. The manual ROI delineation procedure, combined with dedicated fitting algorithms implemented in the MATLAB environment, ensured high accuracy and reproducibility of results, minimizing the impact of limitations inherent in the automatic procedures provided by the MRI system manufacturer.

## 3. Results

### 3.1. Quantitative T_1_ and T_2_ Relaxation Times

Measurement of signal intensity in MRI is a key element in determining T1 and T2 relaxation times, which reflect the dynamics of processes within the tissues studied with MRI. In this case, the signal intensity of the region of interest (ROI) of an atherosclerotic specimen was measured, and the data obtained were used to calculate T1 and T2 relaxation times. These calculations were performed using specialized software that analyzed the changes in signal intensity associated with the relaxation process. For T1, the increase in signal intensity was measured, and for T2, the decay of that signal was measured, allowing the relaxation times for both processes to be calculated accurately.

The T1 relaxation time was determined using the saturation recovery (SR) method, which involves taking a series of measurements using different repetition times (TR) and a fixed echo time (TE). With this method, a varying TR combined with a fixed echo time (e.g., 27 ms) yields a series of T1-weighted images. These images represent how the signal responds to repeated magnetic pulses, giving information about the relaxation processes in the sample under study. The signal intensity (IS) in the ROI region is then measured and stored in DICOM files, which are the standard format for magnetic resonance imaging data. Once the signal intensity data is obtained, the analytical approach is to normalize the data and approximate it using an exponential function. Normalization is the process by which values are converted to a comparative scale to ensure consistency of measurements between different tests and samples. Approximation using an exponential function allows the determination of a characteristic curve that accurately represents the relationship between TR and signal intensity. The T1 time is calculated as the point at which this function reaches 63% of its maximum value. This is the standard definition for relaxation times in the context of MRI, as it indicates the time at which the relaxation process reaches a certain point, which is an important parameter for assessing tissue condition.

Similarly, the T2 relaxation time is calculated using a similar method, except that the TE changes while the TR remains constant. By changing the TE, different characteristics of the relaxation process can be captured, as the echoes at different times will have different intensities depending on the properties of the tissue under study.

**Figure 6:**
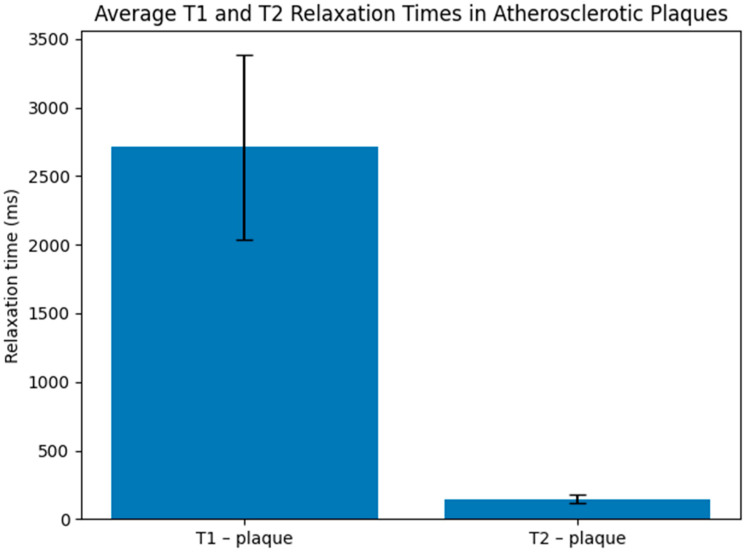
Quantitative T_1_ and T_2_ relaxation times for atherosclerotic plaque samples. Bar chart shows mean relaxation values (T_1_: blue bars; T_2_: orange bars), with error bars representing standard deviation from three repeated measurements. Sample 3 exhibits markedly shorter T_1_ relaxation time (1768 ms) compared to other samples (3196–3294 ms), suggesting compositional heterogeneity.

Figure 6 presents the obtained T_1_ and T_2_ values for the selected samples. The bar chart displays mean relaxation values, with error bars representing standard deviation from three repeated measurements per sample. T_1_ values (blue bars) show considerable variability, with a mean of approximately 2430 ± 620 ms. T_2_ values (orange bars) are less variable, with a mean of 170 ± 25 ms. The observed variation in T_1_ relaxation times reflects compositional heterogeneity within the plaques, potentially related to differences in calcification, lipid content, or the presence of paramagnetic substances. The analysis of the results is presented in the discussion in Section 4.3.

### 3.2. Relaxation Maps

Performing these operations for all image pixels generates a detailed map of the distribution of T1 and T2 relaxation times within the study area. This makes it possible to obtain a spatial map that depicts changes in tissue structure in the atherosclerotic area, which is of great importance in assessing the status and development of atherosclerotic disease. Such maps help diagnose and monitor the progression of the disease, allowing doctors to better understand which areas within the atherosclerotic plaque are more prone to rupture and can lead to serious consequences such as heart attack or stroke.

**Figure 7:**
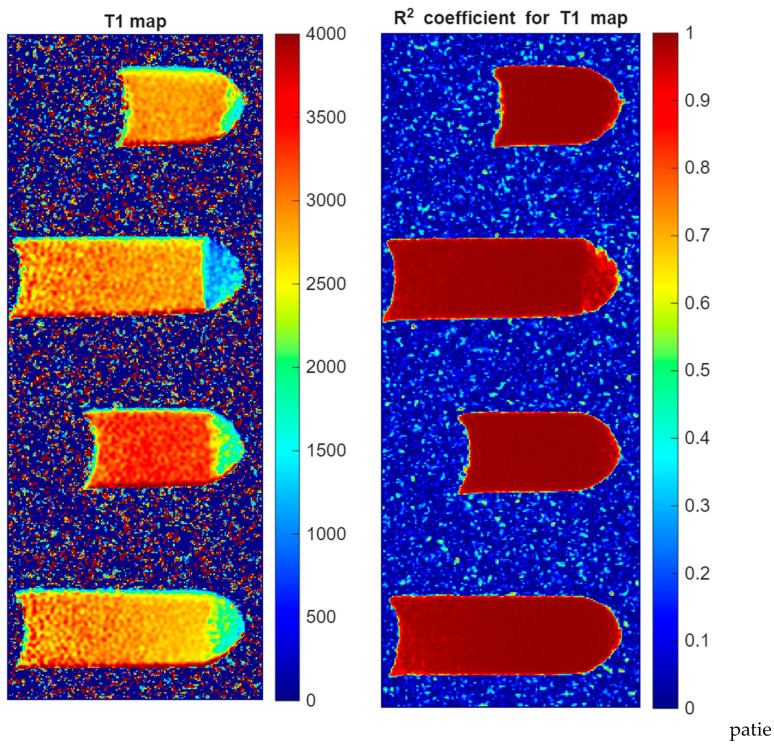
T_1_ relaxation map and R^2^ coefficient map of atherosclerotic plaque specimens. (**Left panel**): Quantitative T_1_ map showing sagittal MRI images of Eppendorf tubes containing plaque samples immersed in water. Color scale indicates T_1_ relaxation time in milliseconds (0–4000 ms). Plaque tissue appears as yellow–orange regions (T_1_ ~ 2500–3500 ms). (**Right panel**): R^2^ coefficient map showing fitting quality from 0 (blue, poor fit) to 1 (red, excellent fit). High R^2^ values (>0.9) within plaque regions confirm reliable T_1_ quantification.

Figure 7 shows the results of the calculations for T_1_ and T_2_ times. The images include samples placed in Eppendorf test tubes, which were used in this study. Imaging was performed in the axial plane, as this orientation was most appropriate given the construction of the measurement system and the surface coil used. Technical and methodological stability was maintained throughout the research process, ensuring the high quality of the results obtained. This figure presents selected tubes from among the tubes examined.

The T_1_ relaxation map displays the spatial distribution of spin–lattice relaxation times across the plaque samples. The color scale represents T_1_ values ranging from 0 to 4000 ms. Plaque tissue appears predominantly as yellow–orange regions corresponding to T_1_ values of approximately 2500–3500 ms. The average T_1_ relaxation time of the plaque tissue was 2963 ± 670 ms, indicating relatively long spin–lattice relaxation typical for heterogeneous biological tissue.

Spatial heterogeneity within the samples is visible, reflecting regional differences in tissue composition, such as lipid cores, fibrous regions, and calcified components. The corresponding R^2^ coefficient map indicates the goodness-of-fit for the relaxation curve fitting at each pixel. High R^2^ values (approaching 1.0, shown in red) within the plaque regions confirm reliable T_1_ measurements. The uniformly high R^2^ values across the plaque areas validate the accuracy of the quantitative relaxation analysis.

The average T_2_ relaxation time of the plaque tissue was 154 ± 33 ms, which is consistent with the expected transverse relaxation behavior of structurally heterogeneous plaque material. Together, the quantitative T_1_ and T_2_ values provide complementary information about the microstructural composition of the analyzed plaque tissue.

**Figure 8:**
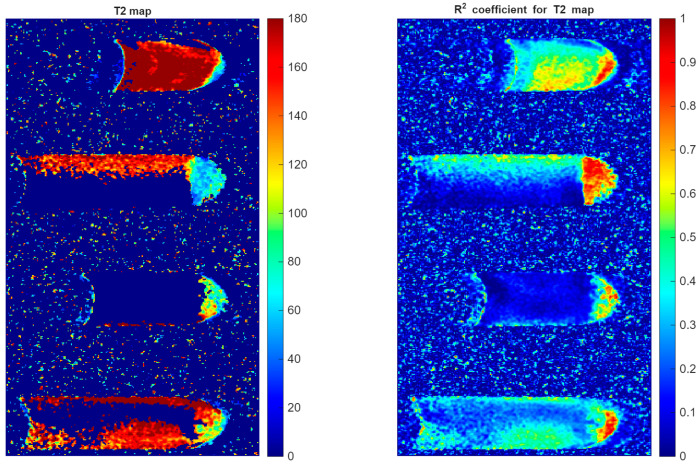
T_2_ relaxation map and R^2^ coefficient map of atherosclerotic plaque specimens. (**Left panel**): Quantitative T_2_ map showing relaxation times (0–180 ms color scale) for plaque samples. (**Right panel**): R^2^ coefficient map indicating curve-fitting quality (0–1 scale). High R^2^ values (yellow–red, >0.6) in plaque regions confirm reliable T_2_ measurements. Regions with low R^2^ (blue, <0.3) were excluded from quantitative analysis to ensure measurement accuracy. The absence of signal in fluid regions reflects noise-related fitting limitations rather than true T_2_ values.

Figure 8 presents the T_2_ relaxation map alongside its corresponding R^2^ coefficient map. This figure shows the same tubes that were presented above; however, in this case, the image displays the distribution of the T_2_ map and the fitting coefficient. T_2_ values within the plaque samples range from approximately 100 to 180 ms.

An explanation is warranted regarding the fluid regions in the Eppendorf tubes. The absence of color signal in the fluid region is related to image noise resulting from the characteristics of the coil used and the imaging conditions in the software that determines T_2_ time. The software intentionally omits values that have a low fitting coefficient, marking these areas with the background color on the T_2_ map. Retaining this data in the image could lead to erroneous conclusions. The low R^2^ coefficient in these regions is clearly visible in the figure. The R^2^ map reveals important regional variations in fitting quality. While plaque tissue generally shows acceptable R^2^ values (indicating reliable measurements), certain regions—particularly at sample edges and within the water compartment—exhibit low R^2^ values (blue regions), reflecting noise-dominated signal where curve fitting is unreliable. The analysis of the results is presented in the discussion in Section 4.3.

### 3.3. Tissue Characterization

On magnetic resonance imaging, intraplaque hemorrhage (IPH) is characterized by high signal in T1-weighted sequences. This phenomenon is due to the presence of methemoglobin, a product of hemoglobin metabolism occurring in bleeding foci within the atherosclerotic plaque. Methemoglobin is formed in the subacute phase of bleeding (usually between days 3 and 7), which allows non-invasive determination of the duration of the pathological process. The presence of IPH, especially with a predominant T1 signal, is an important biomarker of atherosclerotic plaque instability. Numerous studies have shown that intraplaque hematomas correlate with faster progression of atherosclerotic lesions, weakening of the structural integrity of the fibrous cap, and increased risk of vascular incidents such as stroke or transient ischemic attacks (TIA).

Figure 8 presents the T_2_ relaxation map alongside the corresponding R^2^ coefficient map. The figure shows the distribution of T_2_ relaxation times within several plaque samples together with the goodness-of-fit of the relaxation curve fitting. The color scale represents the spatial variation of T_2_ values across the analyzed tissues.

The plaque samples exhibit T_2_ relaxation times in the range of approximately 100–200 ms, with an average value of 154 ± 33 ms. The T_2_ map reveals spatial heterogeneity within the plaque tissue, reflecting local differences in structural composition, such as lipid-rich areas, fibrous regions, and possible calcified components.

The accompanying R^2^ coefficient map demonstrates the reliability of the exponential fitting procedure used to calculate the T_2_ values. High R^2^ values, approaching 1.0 within the plaque regions, indicate a very good agreement between the measured signal decay and the fitted relaxation model, confirming the accuracy of the quantitative T_2_ measurements. Magnetic resonance imaging and characterization of T1 and T2 relaxation times provided important information about the composition and pathomechanisms of atherosclerotic plaque development. IPH, which develops in advanced stages of atherosclerosis, shows high signal in T1-weighted sequences, which is associated with the presence of methemoglobin, a product of hemoglobin degradation that occurs during the subacute phase of bleeding (day 3–7). Methemoglobin, as a paramagnetic substance, increases the T1 signal and can be a non-invasive marker of plaque instability. The measurements showed that T1 and T2 for atherosclerotic plaques are significantly shorter than for pure water, reflecting the presence of complex biological structures that restrict the free movement of protons, such as collagen, inflammatory cells or calcium and iron deposits. The shortening of T2 may additionally be related to the presence of hemosiderin or calcium, while the variation in T1 values may be related to the presence of IPH. As a result, analysis of MR relaxation times is a valuable tool in assessing the structure, activity and potential instability of atherosclerotic plaque (Table 1). The analysis of the results is presented in the discussion in Section 4.4.

### 3.4. Deep Learning-Based Denoising

**Figure 9:**
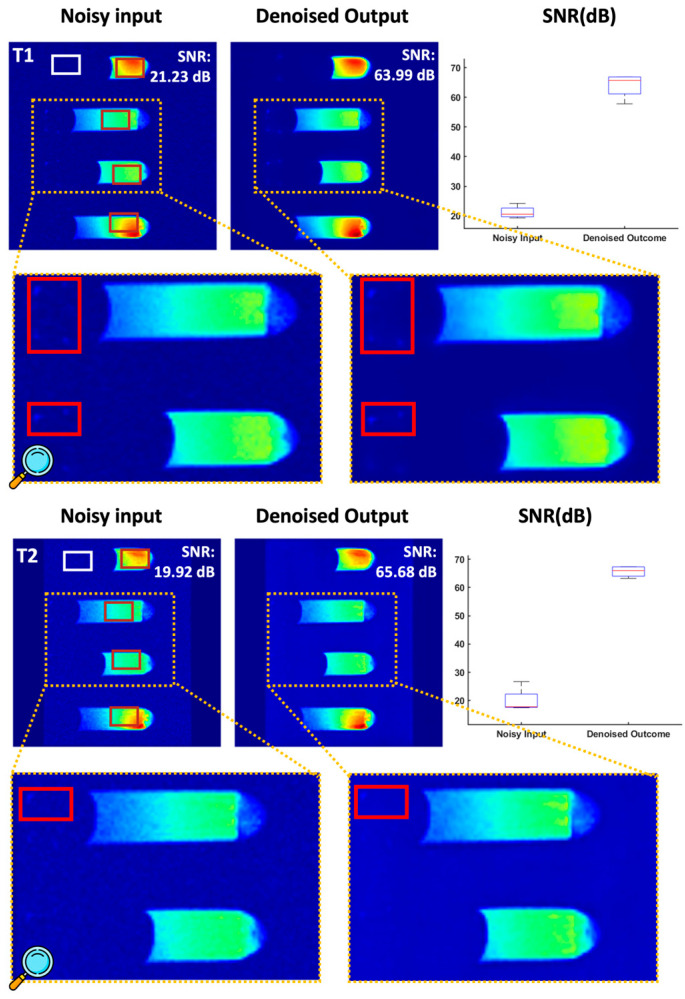
Deep learning-based denoising of T_1_ and T_2_ relaxation maps. (**Top panels**): T_1_ map denoising results showing noisy input (SNR: 21.23 dB) and denoised output (SNR: 63.99 dB) with corresponding box plot demonstrating approximately 3-fold SNR improvement. (**Bottom panels**): T_2_ map denoising showing improvement from 19.92 dB to 65.68 dB. Zoomed insets highlight preservation of anatomical detail and sample boundaries following noise suppression. The Attention–Residual–Dense U-Net architecture effectively reduced Rician noise while maintaining spatial resolution critical for plaque characterization.

The proposed deep learning model achieved substantial noise suppression on Rician-corrupted MRI images while preserving anatomical boundaries and plaque morphology. Visual inspection demonstrated improved delineation of plaque components compared to noisy inputs, particularly in low-signal regions prone to bias in magnitude MRI. Quantitatively, the model yielded reduced mean absolute error (MAE) and improved structural similarity on the test set, as confirmed in Figure 9 Importantly, denoising stabilized signal intensity within regions of interest, which is critical for accurate estimation of T1 and T2 relaxation times. Compared with noisy images, denoised images exhibited 3-fold higher SNR (for both T1 and T2 cases), indicating reduced noise-induced bias. The red boxes are the regions of interest for signals, and the white box is the corresponding noisy background region. We also put up zoomed versions of a specific region, where we can see only noise is removed and the signals are intact even if the intensities are very low (indicated by red rectangles in Figure 9). These improvements directly support more reliable differentiation between lipid-rich, fibrous, hemorrhagic, and calcified plaque components.

## 4. Discussion

This study makes a significant contribution to the development of methods for imaging atherosclerotic plaques by combining quantitative T1 and T2 mapping with advanced deep learning-based noise reduction techniques. Unlike previous studies, which focused primarily on qualitative or semi-quantitative assessment of MRI images, the proposed approach enables a more precise and reproducible characterization of tissue properties.

### 4.1. Clinical Significance and Biological Mechanisms of Atherosclerotic Plaque Development

Understanding the structure and mechanisms of atherosclerotic plaque development is of critical clinical importance, as their composition may be just as significant as the degree of vascular stenosis. Specific morphological features, such as the presence of a lipid component or inflammatory activity, predispose plaques to destabilization, rupture, and thromboembolic complications. Studies of the carotid arteries have shown that plaques rich in lipids and inflammatory components are associated with a higher risk of restenosis after endarterectomy compared to fibrous lesions, which are characterized by greater stability [24,25]. Furthermore, plaque composition may have systemic implications—it has been demonstrated that the characteristics of lesions in one vascular region can predict cardiovascular events in other locations, independent of classical risk factors [26]. Population-based studies further confirm that the presence of intraplaque hemorrhage (IPH) significantly increases the risk of stroke and coronary artery disease, regardless of plaque thickness [27,28]. Micro- and nanoplastics may also represent a new potential risk factor, as their presence in plaques is associated with an increased risk of cardiovascular events [29].

From a biological perspective, the development of an atherosclerotic plaque is a multistage process that begins with endothelial dysfunction, which leads to increased vascular wall permeability and the influx of lipids and inflammatory cells. In subsequent stages, foam cells form, a lipid core develops, and the vessel wall undergoes remodeling. The balance between inflammatory processes and reparative mechanisms determines the stability of the plaque [30,31]. Factors such as hypertension, hyperlipidemia, oxidative stress, and chronic inflammation exacerbate endothelial activation and inflammatory processes, which promotes the progression of atherosclerotic changes [32,33,34]. Persistent inflammation leads to further lipid accumulation, the development of a necrotic core, and vascular remodeling, increasing the risk of plaque destabilization [35,36,37,38].

Vascular smooth muscle cells (VSMCs) also play a significant role in plaque progression and stability; in response to growth and inflammatory factors, they alter their phenotype, increasing their capacity for proliferation, migration, and extracellular matrix production [39,40,41]. This process leads to the formation of a fibrous cap that stabilizes the plaque; however, its disruption may contribute to plaque weakening and rupture [42]. From a clinical perspective, the interaction between the lipid, inflammatory, and structural components of the plaque is particularly important, as it determines its susceptibility to destabilization (Table 2).

### 4.2. MRI Techniques for Atherosclerotic Plaque Characterization

Magnetic resonance imaging has become one of the most valuable non-invasive techniques for evaluating atherosclerotic plaque composition. On MRI, proton density strongly influences signal intensity; calcified tissue with low water content typically appears hypointense, while fibrocellular tissue and thrombus may also exhibit reduced signal intensity on T2-weighted images. T2-weighted imaging enables identification of necrotic cores, although fibrocellular regions containing lipids may also appear hypointense. Partial T2 weighting improves differentiation of plaque components. Diffusion-weighted MRI can detect thrombus and hemorrhage, as thrombi typically appear hyperintense; however, signal interpretation may be affected by nearby calcifications or characteristics of acute clots [52].

MRI is particularly effective in detecting lipid-rich plaque regions and intraplaque hemorrhage (IPH). T1-weighted image contrast depends strongly on acquisition parameters. Fujiwara et al. demonstrated that the mPSIR sequence significantly enhances T1 contrast and improves accuracy of T1 and T2* measurements while providing better IPH visualization [53]. High-field imaging further improves spatial resolution and differentiation of plaque components. Itskovich et al. applied 9.4 T MRI with multicontrast 3D imaging and cluster analysis, achieving high agreement with histopathology [54]. Similarly, Harteveld et al. used 7 T ex vivo MRI with T1, T2, and T2* mapping to demonstrate significant differences in relaxation parameters between fibrous tissue, lipid cores, and calcifications [55]. Coolen et al. performed in vivo studies at 3 T and observed altered relaxation times in carotid plaques compared with healthy tissue, although limited spatial resolution reduced diagnostic precision [56].

Despite these advances, no single MRI sequence can fully distinguish all plaque components [19,57,58,59]. Jiang et al. showed that the fibrous cap exhibits higher signal intensity than the lipid necrotic core in T2-weighted, PD, and STIR images, while calcification appears hypointense in T1-, T2-, and PD-weighted sequences [60]. Best contrast for plaque segmentation was obtained with T2-weighted imaging, whereas T1-weighted sequences have limited ability to differentiate plaque structures in standard clinical FSE sequences [61]. Clinical MRI studies at 1.5 T with dedicated head-and-neck coils allowed precise visualization of carotid stenosis morphology and identification of lipid, fibrous, and calcified plaque components, with strong agreement with histopathology from endarterectomy specimens [62,63,64].

### 4.3. Quantitative Relaxation Mapping and Interpretation of T1 and T2 Values

Quantitative MRI techniques based on relaxation-time mapping provide objective assessment of plaque composition. In the present study, signal intensity measurements within selected regions of interest (ROI) were used to calculate mean T1 and T2 values, which reflect the physicochemical properties of plaque tissues. The mean T1 relaxation time was 2430 ± 620 ms, and the mean T2 relaxation time was 170 ± 25 ms, indicating heterogeneity in plaque composition. Variations likely correspond to differences in lipid-rich regions, collagen-rich fibrous tissue, calcified areas, or paramagnetic substances such as iron or hemosiderin. Shorter T1 and T2 values indicate increased molecular interactions and reduced proton mobility, typical of denser or more mineralized plaques.

These results align with previous studies demonstrating that MRI relaxation times reliably reflect plaque structure. Takaya et al. [10] showed that plaques with intraplaque hemorrhage (IPH) detected by MRI exhibit accelerated progression in plaque and lipid core volume. Yang et al. [65] reported that T1-hyperintense IPH is significantly associated with acute cerebral infarction, supporting the prognostic value of T1 measurements. Ota et al. [66] confirmed that T1-weighted sequences, such as MPRAGE, enable accurate detection of IPH and correlate strongly with histopathology. Treiman et al. [67] further demonstrated that MRI-detected IPH predicts cerebrovascular events and provides more detailed information on plaque stability than luminal stenosis alone.

High-resolution T1 and T2 mapping has also been used to distinguish between plaque components, with mean in vivo T1 values reported as 651 ms for IPH, 1161 ms for necrotic core, and 1447 ms for loose matrix. Shorter T2 values reflect increased molecular interactions and the presence of dense structures or paramagnetic substances, including calcium, iron, or hemosiderin [52]. The heterogeneity observed in the present study likely reflects differences in lipid content, collagen density, and iron-containing hemoglobin degradation products. These findings reinforce the utility of combined T1 and T2 quantitative mapping as a non-invasive tool for assessing plaque composition, detecting hemorrhagic components, and identifying plaques at higher risk for progression and cerebrovascular events [68,69].

The lack of histological validation means that these associations remain speculative.

### 4.4. Physicochemical Processes and Experimental Plaque Models

Experimental studies often use water or blood-mimicking fluids to investigate the mechanical properties of atherosclerotic plaques and vessel wall behavior. Such models allow controlled assessment of plaque deformation and rupture risk and can be combined with imaging techniques, including MRI, CT, and Doppler ultrasound [70,71]. Interactions between water, lipids, and proteins within plaques depend on tissue composition and structure, influencing diffusion properties and MRI signal characteristics. In particular, lipid-rich regions exhibit restricted diffusion, while mineral deposits, primarily hydroxyapatite, alter mechanical properties and reduce vascular elasticity [72,73,74].

**Figure 10:**
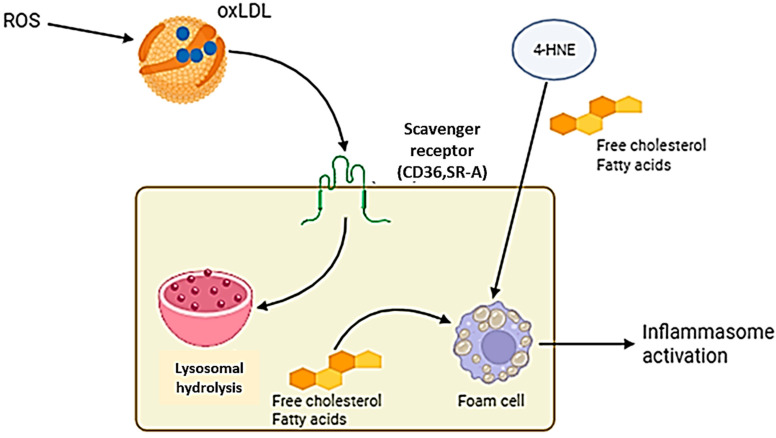
Mechanism of foam cell formation through scavenger receptor-mediated uptake of oxidized LDL. Reactive oxygen species (ROS) oxidize native LDL to form oxLDL, which is recognized by scavenger receptors (CD36, SR-A) on macrophages. Unlike classical LDL receptors, scavenger receptors lack negative feedback regulation, leading to uncontrolled lipid uptake. Lysosomal hydrolysis releases free cholesterol and fatty acids, while lipid metabolites such as 4-hydroxynonenal (4-HNE) contribute to inflammasome activation. Progressive lipid accumulation transforms macrophages into foam cells, a hallmark of early atherosclerotic lesions.

Foam cell formation is one of the earliest stages in plaque development (Figure 10). LDL undergoes oxidative modification and is taken up by macrophages, leading to foam cell accumulation and lipid core formation [75,76]. These processes activate inflammatory pathways and contribute to extracellular matrix remodeling, which weakens the fibrous cap and increases plaque vulnerability [77,78]. Oxidative stress further affects plaque stability through protein and ECM modifications, including reactions involving reactive nitrogen species [79].

The interplay of lipid accumulation, inflammation, oxidative stress, and ECM remodeling underlies plaque progression and is directly reflected in MRI measurements. Variations in lipid content, collagen density, calcifications, and hemorrhagic components influence relaxation times, contributing to the heterogeneity observed in T1 and T2 mapping [10,52,68,69].

### 4.5. Influence of Noise and Deep Learning Denoising on Quantitative MRI

In this study, the proposed deep learning-based denoising model significantly improved image quality in MRI datasets contaminated with Rician noise, particularly in areas with low signal intensity. Visual analysis revealed better representation of atherosclerotic plaque components after denoising, and quantitative metrics confirmed a threefold increase in the signal-to-noise ratio (SNR) for T1 and T2 cases compared to noisy images, supporting a more reliable assessment of relaxation parameters. This improvement is crucial in quantitative MRI, as traditional denoising methods often result in the loss of spatial detail and introduce a blurring effect that can obscure microstructural features of interest. Deep learning-based approaches can model complex noise distributions, such as Rician noise in MRI, and suppress noise while preserving structural boundaries, resulting in better SNR and structural similarity metrics compared to traditional filters, e.g., non-local means [80].

An important aspect is that deep learning-based denoising can preserve quantitative MRI markers, including T1 and T2 relaxation times, while improving image quality and reducing acquisition time. For example, in a clinical study of brain tumors, the deep learning reconstruction (DLR) algorithm preserved the mean T1 and T2 relaxation times and generated relaxation maps with higher structural similarity compared to long acquisition sequences, demonstrating that denoising does not distort the biological information contained in quantitative measurements [81]. These results are consistent with the observations from the present study, in which denoised images not only improved visual quality but also stabilized signal intensity in the ROI, which is crucial for fitting relaxation curves.

Accurate quantitative MRI analysis of atherosclerotic plaques requires high-quality images and adequate SNR. In MRI magnitude images, noise follows a Rayleigh distribution resulting from Gaussian disturbances in the complex signal domain, which introduces error in areas with low SNR and affects the estimation of relaxation parameters [82]. Traditional denoising methods can reduce noise but often cause blurring that obscures fine, clinically relevant plaque features. Modern approaches based on neural networks, trained on simulated Rician noise, effectively suppress noise while preserving key structural details [83,84]. This study employed a deep learning framework utilizing residual learning, dense feature propagation, and attention mechanisms, which improved the quality of MRI images and the stability of relaxation time estimates, enabling the detection of subtle plaque components, including intraplaque hemorrhages, thin fibrous caps, and heterogeneous tissue structure.

Although the denoising network was trained using a brain tumor MRI dataset, the task formulation in this study is fundamentally a low-level image restoration problem, where the objective is to learn a mapping between noisy and high SNR images. In MRI, noise in magnitude images follows a Rician distribution, which arises from the underlying acquisition physics and is largely independent of the anatomical structure being imaged. Consequently, the statistical properties of noise remain consistent across different tissues and imaging targets acquired under similar scanner conditions (e.g., 1.5 T field strength). Our model is designed to exploit local spatial correlations, multi-scale feature aggregation, and residual learning, which collectively promote robustness to domain shifts in anatomical content while preserving sensitivity to noise characteristics. This enables the model to generalize beyond the specific structures present in the training dataset.

Furthermore, our prior work has demonstrated that deep learning-based denoising models trained on structurally dissimilar datasets, including inanimate phantoms, can generalize effectively to biological imaging scenarios and across different modalities, provided that the noise statistics are comparable [20,21,22,23]. Consistent with these findings, the application of the trained model to atherosclerotic plaque MRI in the present study resulted in a threefold improvement in SNR while preserving microstructural features, suggesting that the learned mapping is governed by noise characteristics rather than anatomical priors. Nevertheless, we acknowledge that incorporating domain-specific training data or fine-tuning strategies could further enhance robustness, and this represents an important direction for future work.

Also note that our model was trained exclusively on a publicly available brain tumor MRI dataset and applied directly to ex vivo atherosclerotic plaque images without any domain-specific fine-tuning or additional validation on plaque-specific data. This cross-domain transfer relies on the premise that Rician noise statistics—arising from Gaussian perturbations in the complex MR signal domain—are largely independent of the underlying anatomy and remain consistent across different tissues and coil configurations at the same field strength. Moreover, the denoising task was framed as a low-level image restoration problem, where the network learns a mapping from noisy to clean magnitude images based on local spatial correlations and multi-scale features without requiring high-level semantic understanding of plaque morphology. While the observed threefold improvement in SNR and preservation of microstructural details support the generalizability of this approach, we explicitly acknowledge that no dedicated validation was performed on atherosclerotic plaque data—neither through independent test sets from the same domain nor via fine tuning on plaque-specific noise characteristics. Consequently, the quantitative T1 and T2 values reported after denoising should be interpreted with this limitation in mind. Future work will focus on acquiring paired noisy–clean plaque datasets and assessing model performance with plaque-specific metrics, including histological correlation, to fully validate the clinical translatability of the method.

### 4.6. Implications for Imaging Biomarkers and Cardiovascular Risk Prediction

The results of this study confirm that quantitative magnetic resonance parameters, particularly T1 and T2 relaxation times, reflect the morphological and biochemical properties of atherosclerotic plaques. These parameters serve as an indirect indicator of the tissue composition of atherosclerotic lesions, allowing for the differentiation of key plaque components, such as the lipid core, fibrous areas, calcifications, and intraplaque hemorrhages. Changes in T1 and T2 values result from differences in tissue microstructure, water content, lipids, hemoglobin degradation products, and the density of collagen and other extracellular matrix components.

A key element of this study was the use of deep learning-based denoising methods, which improved the quality of MRI images and stabilized the estimation of relaxation parameters. In MRI images with a low signal-to-noise ratio (SNR), the presence of Rician noise can lead to significant signal distortion, especially in low-intensity regions, which hinders reliable quantitative analysis. Modern denoising algorithms enable effective noise reduction while preserving structural details, which is crucial for detecting subtle morphological features of the plaque, such as a thin fibrous cap or small hemorrhagic foci.

The combination of advanced quantitative analysis of relaxation parameters with image enhancement techniques significantly enhances the diagnostic capabilities of MRI in the assessment of atherosclerosis. A more precise characterization of plaque composition enables the identification of so-called rupture-prone plaques, which are associated with an increased risk of cardiovascular events, such as stroke or myocardial infarction. Lipid-rich areas and necrotic cores exhibit different relaxation properties than fibrous tissue or calcifications, as do intralaminar hemorrhages associated with methemoglobin and hemoglobin degradation products, which cause characteristic changes in relaxation parameters. Therefore, T1 and T2 mapping can serve as a valuable, non-invasive tool for assessing the composition and severity of atherosclerotic lesions and support precise clinical risk stratification.

An important aspect of the clinical translation of these findings is the potential integration of atherosclerotic plaque characterization into existing cardiac magnetic resonance imaging (CMR) protocols. Currently, CMR is widely used to assess myocardial structure and function, including the detection of ischemic injury and fibrosis using late gadolinium enhancement (LGE). It should be emphasized, however, that these methods primarily reflect the consequences of coronary artery disease rather than its underlying cause.

Incorporating the assessment of atherosclerotic plaque composition into CMR imaging could provide complementary information, enabling the simultaneous evaluation of both myocardial damage and the vascular changes responsible for ischemia. In particular, the identification of high-risk plaque features, such as a lipid necrotic core or intraplaque hemorrhage, may provide additional prognostic value beyond the characteristics of the myocardial tissue alone. Previous studies suggest that the combination of vascular and myocardial imaging may improve risk stratification and better predict adverse cardiovascular events [85].

Despite this potential, the implementation of such an approach into routine clinical practice faces significant technical and methodological limitations. Coronary artery imaging remains constrained by spatial resolution, cardiac and respiratory motion, and long acquisition times. Additionally, differences between imaging of large vessels (e.g., carotid arteries) and coronary arteries—including their size, mobility, and the complexity of surrounding structures—can significantly impact the ability to reliably assess plaques. Standardization of imaging protocols and validation of quantitative MRI biomarkers are also necessary [86].

Notwithstanding these limitations, the integration of quantitative atherosclerotic plaque imaging with myocardial tissue assessment represents a promising direction for further research and may contribute to a more comprehensive and personalized assessment of cardiovascular risk.

### 4.7. Limitations of This Study and the Potential Complementarity of MRI and Other Imaging Techniques

This study did not include a direct comparison of MRI results with other imaging methods, such as computed tomography (CT) or ultrasound, due to the experimental nature of the work and the use of ex vivo material. It is worth noting, however, that these techniques provide complementary information—CT is particularly sensitive in detecting calcifications, while MRI allows for a more detailed assessment of the tissue composition of atherosclerotic plaques, including the presence of lipid cores or intraplaque hemorrhages.

The relaxation parameters obtained cannot be directly extrapolated to in vivo conditions, where imaging quality depends on factors such as patient motion, blood flow, or spatial resolution limitations. This constitutes the main translational limitation of this study. Nevertheless, ex vivo analysis allows for precise control of measurement conditions and accurate assessment of tissue properties, which constitutes a crucial step in the validation of quantitative imaging methods.

Despite these limitations, the obtained results can provide a basis for designing future in vivo studies, particularly those utilizing a multimodal approach. Combining MRI with other imaging techniques has the potential to improve characterization of the composition and properties of atherosclerotic plaques, but this requires further validation in a clinical setting. Integrating data from different imaging modalities may support the development of more advanced diagnostic strategies in the future, but its relevance for cardiovascular risk stratification remains unclear at this stage.

Furthermore, this study did not correlate with histopathological examination, limiting the ability to clearly interpret relaxation times in the context of plaque composition.

## 5. Conclusions

Measuring signal intensity in MRI allows determination of T1 and T2 relaxation times, which reflect tissue properties. In this study, atherosclerotic specimens were analyzed by measuring signal intensity in ROIs and determining T1 and T2 using specialized software. For T1, variable TR with constant TE was used, and for T2, constant TR and variable TE were used. The results were presented on images in the sagittal plane. Calculations were based on normalization and exponential approximation, and relaxation times were determined from characteristic curve points.

IPHs show a high T1 signal due to the presence of methemoglobin, making them a potential marker of plaque instability. T1 and T2 values in the samples were lower than in water, indicating the presence of complex biological structures, such as collagen, inflammatory cells, calcium and iron. The differences between the samples reflect their heterogeneity, and the shorter T1 and T2 times may suggest greater calcification or the presence of hemosiderin. MRI provides information about the composition and stability of the atherosclerotic plaque, which is more clinically relevant than its size. The presence of IPH is associated with a higher risk of myocardial infarction and stroke, independent of other factors. The composition of plaques in the carotid arteries may also predict the risk of cardiovascular events in other areas. Micro- and nanoplastics, present in 58% of samples, are a new risk factor and are associated with a higher risk of vascular complications.

MRI is also a tool in biomechanical analysis. Water as a medium allows the mechanical properties of plaques and blood flow to be studied, although it does not reproduce all blood characteristics. T2-weighted images and parametric techniques, such as T2 mapping or mPSIR sequences, increase diagnostic accuracy. Analysis of relaxation times allows not only assessment of plaque composition but also risk prediction and treatment planning.

Moreover, this study demonstrates that deep learning–based denoising with explicit Rician noise modeling can substantially improve the quality and quantitative reliability of MRI images used for atherosclerotic plaque analysis. By increasing SNR and reducing signal-dependent magnitude bias, the proposed Attention–Residual–Dense U-Net stabilizes voxel-wise signal intensities and improves the robustness of T1 and T2 relaxation-time estimation—key biomarkers for plaque composition and vulnerability assessment. The integration of physics-aware noise simulation, structural-similarity-driven optimization, and advanced neural network architectures provides a robust framework for quantitative MRI enhancement. Such approaches have strong potential to improve non-invasive identification of high-risk plaques, reduce uncertainty in quantitative biomarkers, and facilitate the translation of advanced MRI techniques into routine clinical and research workflows.

## Figures and Tables

**Table 1:** MRI tissue characterization of atherosclerotic plaque components based on T_1_ and T_2_ relaxation properties.

Interpretation	Relationship to T1/T2	Atherosclerotic Mechanism	Aspect
Plaque instability marker	Elevated T1 in T1-weighted sequences	Conversion of hemoglobin to methemoglobin	IPH (hemorrhaging)
Advanced atherosclerosis	Reduced T2	Necrosis, a chronic process	Calcifications, iron
Reduced proton mobility	Reduced T1 and T2	Vascular remodeling	Fibrous tissue, collagen
Reference standard	High T1 and T2	Lack of cellular structures	Water (control)

**Table 2:** Molecular and cellular composition of atherosclerotic plaque. Summary of the major structural components organized by category: lipids (free cholesterol, cholesterol esters, triglycerides, phospholipids), cells (macrophages, T lymphocytes, smooth muscle cells, endothelial cells), extracellular matrix (collagen types I and III, elastin, proteoglycans), inflammatory factors (interleukins IL-1β/IL-6, TNF-α, MCP-1), proteolytic enzymes (metalloproteinases MMP-2/MMP-9), and other components (cholesterol crystals, calcium, hemoglobin/hemosiderin). Each component’s role in plaque development, stability, and destabilization is described [24,43,44,45,46,47,48,49,50,51].

Description/Meaning	Component	Component Group
Form lipid core, can crystallize and destabilize plaque Stored in macrophages (foam cells) and lipid core Occur in smaller amounts, indicate metabolic disorders They are part of cell membranes and lipoproteins	Free cholesterol Cholesterol esters Triglycerides Phospholipids	Lipids
Phagocytose lipids, produce pro-inflammatory cytokines, key in the development of inflammation Regulate the immune response They form a fibrous cap, stabilize the lamina Regulate the permeability and influx of cells into the vessel wall	Macrophages T lymphocytes Smooth muscle cells Endothelial cells	Cells
Give structure to the fibrous cap of the plaque Promote vascular elasticity Facilitate retention of lipoproteins	Collagen types I and III Elastin Proteoglycans	Extracellular matrix
Promote inflammation and destabilization of plaque Strongly pro-inflammatory, activate endothelial cells and macrophages Attract monocytes to the vessel wall	Interleukins (e.g., IL-1β, IL-6) TNF-α MCP-1	Inflammatory factors
Degrade collagen and extracellular matrix, weakening the fibrous cap	Metalloproteinases (MMP-2, MMP-9)	Proteolytic enzymes
Cause mechanical damage and activation of the inflammasome Deposit in later stages, can stabilize or stiffen the plaque Present with intraplaque bleeding	Cholesterol crystals Calcium Hemoglobin/hemosiderin	Other

## Data Availability

The original contributions presented in this study are included in the article. Further inquiries can be directed to the corresponding author.

## Abbreviations

The following abbreviations are used in this manuscript:

3D TOF	Three-Dimensional Time-of-Flight
ATP	Adenosine Triphosphate
CD36	Cluster of Differentiation 36
CHD	Coronary Heart Disease
CRP	C-Reactive Protein
CT	Computed Tomography
EDTA	Ethylenediaminetetraacetic Acid
EGF/EGFR	Epidermal Growth Factor/Epidermal Growth Factor Receptor
eNOS/iNOS	Endothelial Nitric Oxide Synthase/Inducible Nitric Oxide Synthase
ERK	Extracellular Signal-Regulated Kinase
FGF	Fibroblast Growth Factor
FGFR	Fibroblast Growth Factor Receptor
HGF	Hepatocyte Growth Factor
HIF	Hypoxia-Inducible Factor
HR	Heart Rate
ICAM-1	Intercellular Adhesion Molecule 1
IFN-γ	Interferon Gamma
IL-1β	Interleukin-1 Beta
IL-6	Interleukin-6
IPH	Intraplaque Hemorrhage
IVUS	Intravascular Ultrasound
LC3	Microtubule-Associated Protein 1A/1B-Light Chain 3
LDL	Low-Density Lipoprotein
LM	Left Main (Coronary Artery)
LME	Left Main Equivalent
LRNC	Lipid-Rich Necrotic Core
MCP-1	Monocyte Chemoattractant Protein-1
MDA	Malondialdehyde
MMP(x)	Matrix Metalloproteinase (various isoforms)
MMP	Matrix Metalloproteinase
MNPs	Magnetic Nanoparticles
MPO	Myeloperoxidase
mPSIR	Modified Phase-Sensitive Inversion Recovery
MRI	Magnetic Resonance Imaging
NADPH	Nicotinamide Adenine Dinucleotide Phosphate (reduced form)
NC	Necrotic Core
NF-κB	Nuclear Factor Kappa B
NOX2	NADPH Oxidase 2
nSMase2	Neutral Sphingomyelinase 2
oxLDL	Oxidized Low-Density Lipoprotein
P2RY12	Purinergic Receptor P2Y12
PD	Proton Density
PDGF	Platelet-Derived Growth Factor
PDGF/PDGFR	Platelet-Derived Growth Factor/Platelet-Derived Growth Factor Receptor
PI3K–AKT	Phosphoinositide 3-Kinase–Protein Kinase B Signaling Pathway
Pro-MMP	Pro-Matrix Metalloproteinase
ROI	Region of Interest
ROS	Reactive Oxygen Species
S1P/S1PR	Sphingosine-1-Phosphate/Sphingosine-1-Phosphate Receptor
SQSTM1	Sequestosome 1
STIR	Short Tau Inversion Recovery
T1	Longitudinal Relaxation Time
T2	Transverse Relaxation Time
TGF-β	Transforming Growth Factor Beta
TGF-β/TGFβR	Transforming Growth Factor Beta/Transforming Growth Factor Beta Receptor
TIA	Transient Ischemic Attack
TK	Tyrosine Kinase
TNF-α	Tumor Necrosis Factor Alpha
TR	Repetition Time
TSP-1	Thrombospondin-1
VCAM-1	Vascular Cell Adhesion Molecule 1
VEGF	Vascular Endothelial Growth Factor
VEGF/VEGFR	Vascular Endothelial Growth Factor/Vascular Endothelial Growth Factor Receptor
VSMCs	Vascular Smooth Muscle Cells
